# Risk factors analysis and nomogram model construction for postoperative atrial fibrillation after off-pump coronary artery bypass grafting

**DOI:** 10.12669/pjms.41.1.11087

**Published:** 2025-01

**Authors:** Long Qian, Chuanxian Hu, Mengtao Fan, Zhuqing Ji

**Affiliations:** 1 Long Qian Department of Cardiothoracic Surgery, The Affiliated Huai’an 1st People’s Hospital of Nanjing Medical University, Huaian, Jiangsu Province 223300, P.R. China; 2 Chuanxian Hu Department of Cardiothoracic Surgery, The Affiliated Huai’an 1st People’s Hospital of Nanjing Medical University, Huaian, Jiangsu Province 223300, P.R. China; 3 Mengtao Fan Department of Cardiothoracic Surgery, The Affiliated Huai’an 1st People’s Hospital of Nanjing Medical University, Huaian, Jiangsu Province 223300, P.R. China; 4 Zhuqing Ji Department of Medicine Oncology, The Affiliated Huai’an 1st People’s Hospital of Nanjing Medical University, Huaian, Jiangsu Province 223300, P.R. China

**Keywords:** Postoperative atrial fibrillation, Off-pump coronary artery bypass grafting, Nomogram predictive model, Risk factors

## Abstract

**Objective:**

To explore the risk factors associated with postoperative atrial fibrillation (POAF) after off-pump coronary artery bypass grafting (OPCABG) and to construct a nomogram predictive model.

**Methods:**

In this retrospective cohort study, clinical data of 193 patients who received OPCABG in Huai’an First People’s Hospital Affiliated to Nanjing Medical University from June 2021 to November 2023 were retrospectively analyzed. Based on the established diagnosis of POAF, patients were divided into the POAF group (n=75) and the non-POAF group (n=118). Logistic regression analysis was used to screen for risk factors for POAF after OPCABG. A nomogram prediction model for POAF after OPCABG was constructed based on the independent risk factors, and the model was validated by calibration curve and the area under the receiver operating characteristic curve (AUC).

**Results:**

The incidence of POAF after OPCABG in the cohort was 38.86% (75/193). Age, diabetes, history of percutaneous coronary intervention (PCI), duration of operation, length of hospital stay and *left ventricular ejection fraction* (*LVEF*) were identified as independent risk factors for POAF after OPCABG (*P*<0.05). The concordance index of the nomogram model for predicting the risk of after POAF after OPCABG based on the six independent risk factors was 0.820. The correction curve tended towards the ideal curve, and the area under the receiver operating characteristic curve was 0.820 (95% CI:0.758~0 882).

**Conclusions:**

Age, diabetes, history of PCI, duration of operation, length of hospital stay and LVEF are independent risk factors for POAF after OPCABG. The constructed nomogram model has a good predictive performance for predicting POAF in patients after OPCABG.

## INTRODUCTION

Coronary artery bypass grafting (CABG) after cardiac arrest under extracorporeal circulation (EC) is considered one of the primary surgical methods of revascularization in patients with coronary heart disease.^1^,^2^ However, EC is associated with high risk of complications such as surgical trauma, ischemia-reperfusion injury, and systemic inflammatory response syndrome.^3^ Off-pump coronary artery bypass grafting (OPCABG) is a less invasive approach that avoids cardiopulmonary bypass by using a cardiac surface fixator, thus lowering the risks of EC-associated adverse effects.^1^,^2^,^4^

Postoperative atrial fibrillation (POAF) is a common complication after CABG. Studies have shown that POAF may increase the risk of stroke, prolong hospital stay, increase treatment costs, and is associated with high mortality rates and poorer outcomes.^5^–^7^ While OPCABG may reduce the risk of POAF^7^, some patients may still develop atrial fibrillation after the surgery. Therefore, the occurrence of POAF in patients who undergo CABG without EC may be influenced by additional factors.^8^,^9^ POAF may seriously affect hemodynamic stability, and increase the incidence of thrombotic events in patients, as well as prolong overall hospitalization in the intensive care unit (ICU), and increase the associated medical burden.^8^–^10^ Therefore, timely prevention of POAF after OPCABG is crucial.

Nomograms have been widely used in medical research for predicting the probability of clinical outcomes. In recent years, studies have established nomogram model to predict POAF after CABG.^11^,^12^ Although the currently established nomogram models have shown good predictive performance, models with improved predictive accuracy are still required. Therefore, this study aimed to identify independent risk factors for POAF after OPCABG, and to construct a novel nomogram model that may provide improved accuracy for predicting clinical outcomes^13^ and potentially provide references for clinicians.

## METHODS

In this retrospective cohort study, medical records of 193 patients (132 males and 61 females) who underwent OPCABG in Huai’an First People’s Hospital Affiliated to Nanjing Medical University from June 2021 to November 2023 were retrospectively analyzed.

### Ethical Approval:

The ethics committee of our hospital approved the study on April 8^th^ 2024, number: KY-2024-052-01.

### Inclusion criteria:

- Patients with coronary heart disease who underwent OPCABG alone without any other complications.^2^,^4^ Peshawar over a duration of 15 months (December 2020 - March 2022
- Patients did not require extracorporeal circulation, with continuous heartbeat through the entire OPCABG procedure.
- Complete clinical data.
- The NYHA classification of cardiac function: grades I-III.
- Patients older than 18 years.

### Exclusion criteria:

- Patients with a history of POAF before OPCABG.
- Patients who underwent congenital heart disease repair and valve replacement surgery during OPCABG.
- Preoperative sudden myocardial infarction patients.
- Patients with a history of congestive heart failure.
- Patients with valve stenosis.
- Patients who have previously undergone pacemaker implantation treatment.

### POAF definition:

Any atrial fibrillation that requires treatment during the perioperative period, with each atrial fibrillation lasting ≥ 20 minutes and a cumulative duration of atrial fibrillation lasting ≥ 60 minutes within 24 hours.^14^ According to the definition of POAF, patients were divided into the POAF group or the Non-POAF group.

### Data collection:

Following relevant information was collected from all patients:

- General information, including gender, age, body mass index (BMI), smoking status, and alcohol consumption.
- Basic diseases: including hypertension, diabetes, and a history of percutaneous coronary intervention (PCI).
- Perioperative characteristics including duration of operation and length of hospital stay.
- Laboratory examination indicators including serum creatinine (Scr), blood urea nitrogen (BUN), triglycerides (TG), total cholesterol (TC) levels, prothrombin time (PT), activated partial thromboplastin time (APTT), troponin, etc. Levels of Scr, BUN, TG, and TC were measured using AU-2700 fully automatic biochemical analyzer (Japan). PT and APTT levels were detected using Sysmex CA-8000 fully automatic coagulation analyzer (Japan). Troponin was measured using the ELX-800 enzyme-linked immunosorbent assay reader (BioTek, US).
- Cardiovascular indicators including the number of vascular lesions, left atrial size, and left ventricular ejection fraction (LVEF). All parameters were measured using the Philips IE33 echocardiography.

### Statistical Analysis:

SPSS 26.0 (IBM Corp, Armonk, NY, USA) was used for analysis. Continuous data were expressed as mean ± standard deviation, and independent sample t-test was used for inter group comparison. Counting data were presented as frequency and composition ratio (%), and Chi square test was used for comparison between groups. Logistic regression analysis was conducted to analyze the risk factors for POAF after OPCABG. A nomogram model was constructed based on the identified risk factors using RMS package in the R software (R3.5.3). Concordance index (C-index) was calculated using the RMS package. Calibration curves and receiver operating characteristic (ROC) curves were drawn to evaluate the predictive performance of the model. *P*<0.05 indicated a statistically significant difference.

## RESULTS

**Fig.1:**
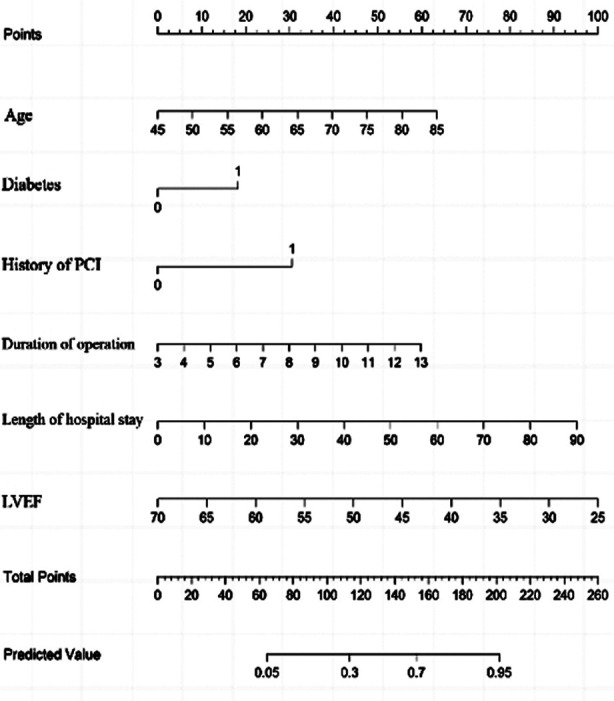
Nomogram prediction model for POAF after OPCABG.

**Fig.2:**
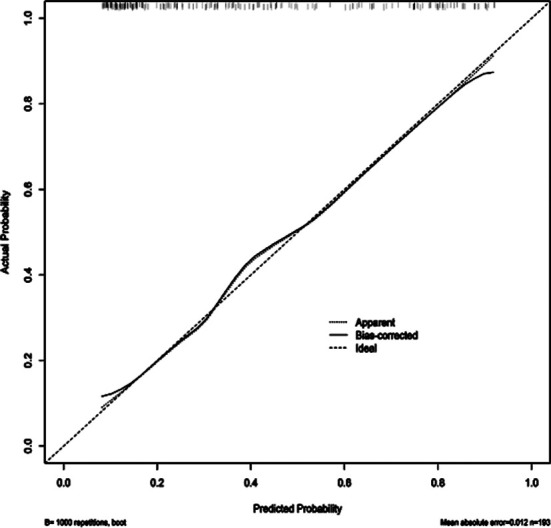
Calibration curve.

**Fig.3:**
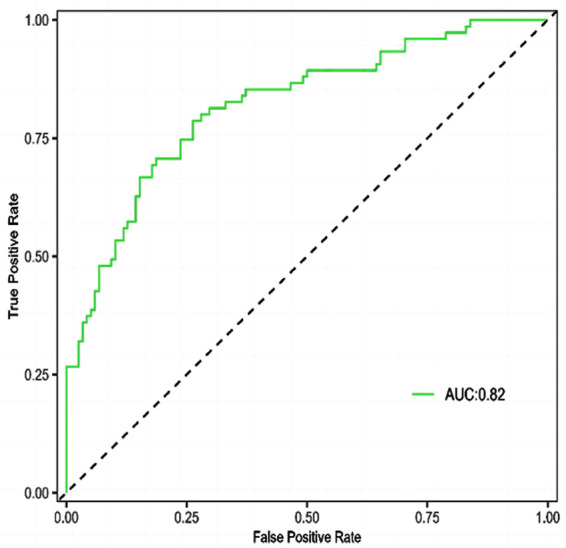
ROC curve.

Of 193 patients with OPCABG, 75 experienced POAF, with an incidence of 38.86%. There were statistically significant differences in age, diabetes, history of PCI, duration of operation, length of hospital stays, troponin level and LVEF level between the two groups (*P*<0.05) (Table-I). Logistic regression analysis showed that age, diabetes, history of PCI, duration of operation, length of hospital stay, LVEF, and troponin were all independent risk factors for POAF after OPCABG (*P*<0.05) (Table-II). Based on the above six independent risk factors, a nomogram model for predicting the risk of POAF in patients after OPCABG was constructed (Fig.1). The model validation results showed the C-index of 0.820, indicating that the nomogram model has a good discrimination. The calibration curve validation results of the nomogram model showed that the predicted values were basically consistent with the measured values, and the calibration curve approached the ideal curve, indicating good prediction accuracy of the model (Fig.2). The ROC curve validation results of the nomogram model showed the area under the ROC curve (AUC) of 0.820 (95% CI: 0.758-0.882) (Fig.3).

**Table-I:** Univariate analysis of POAF in patients after OPCABG.

Variable	POAF group (n=75)	Non-POAF group (n=118)	χ^2^/t	P
Male (yes)	54 (72.00)	78 (66.10)	0.738	0.390
BMI (kg/m^2^)	23.26±2.95	23.41±2.98	0.321	0.748
Hypertension (yes)	43 (57.33)	76 (64.41)	0.971	0.325
Age (year)	67.31±7.67	63.77±8.06	-3.027	0.003
Diabetes (yes)	38 (50.67)	32 (27.12)	11.000	0.001
History of PCI (yes)	19 (25.33)	16 (13.56)	4.282	0.039
Smoking (yes)	21 (28.00)	36 (30.51)	0.139	0.710
Alcohol consumption (yes)	19 (25.33)	35 (29.66)	0.426	0.514
Duration of operation (hour)	6.09±1.73	5.53±1.36	-2.504	0.013
Length of hospital stay	27.45±8.37	22.20±9.79	-3.837	<0.001
Scr (umol/L)	101.73±12.67	98.18±13.51	1.822	0.070
BUN (mmol/L)	9.31±1.97	8.97±2.35	1.042	0.299
TG (mmol/L)	3.59±1.12	3.47±1.08	0.742	0.459
TC (mmol/L)	3.72±1.35	3.96±1.24	1.266	0.207
PT (s)	13.54±2.91	12.80±3.06	1.669	0.097
APTT (s)	31.90±5.91	33.15±6.22	1.387	0.167
Troponin (ug/L)	0.76±0.35	0.65±0.31	-2.149	0.033
Number of vascular lesions (piece)	2.48±0.67	2.59±0.71	1.072	0.285
Left atrial size (mm)	33.91±4.05	35.09±6.32	1.439	0.152
LVEF (%)	50.51±9.79	58.17±7.64	5.758	<0.001

**Table-II:** Analysis of risk factors for POAF after OPCABG.

Variable	β	S.E.	Wald χ^2^	P	OR	95%CI
Age	0.067	0.023	8.422	0.004	1.069	1.022~1.118
Diabetes	0.782	0.378	4.28	0.039	2.186	1.042~4.586
History of PCI	1.303	0.476	7.5	0.006	3.679	1.448~9.346
Duration of operation	0.264	0.123	4.595	0.032	1.302	1.023~1.658
Length of hospital stay	0.046	0.019	5.743	0.017	1.047	1.008~1.086
LVEF	-0.092	0.022	17.89	<0.001	0.912	0.874~0.952
Troponin	0.645	0.575	1.258	0.262	1.905	0.618~5.876
Constant	-3.472	2.161	2.581	0.108	0.031	

## DISCUSSION

This study showed that 38.86% (75/118) of patients developed POAF after OPCABG, further confirming that the incidence of POAF still remains high in our hospital. Li B et al.^15^ analyzed clinical data of 362 patients who underwent OPCABG, and found that 28.7% (104/362) of them developed POAF. The study of Lei L et al.^16^ showed that the incidence of POAF after OPCABG was 16.9% (40/237), while Lewicki L et al.^17^ reported that the incidence of POAF after OPCABG is 19.3%. The incidence of POAF in our study is much higher than in the previous studies, which may be related to sample size and potential selection bias. These results indicate that reducing the incidence of POAF should still remain the focus of our hospital’s work.

In addition, the current study showed that age, diabetes history, history of PCI, duration of operation, length of hospital stays, and LVEF are all independent risk factors of POAF after OPCABG. Advanced age is a recognized risk factor for POAF after OPCABG.^18^,^19^ Luo W et al.^18^ found that the age of patients with POAF after OPCABG is significantly higher than that of the non POAF group. A study by Zhang R et al.^19^ that included 61 elderly patients and 585 young patients receiving OPCABG for prognostic factor analysis confirmed that elderly patients are more prone to adverse events such as POAF after OPCABG, and the risk of POAF increases by 75% for every 10 years of age. We may speculate that the advanced age is associated with increased degree of myocardial fibrosis, leading to sinoatrial node fibrosis, increased lipid content, and amyloid deposition in the myocardial interstitium. These factors are all important pathological foundations of POAF. Therefore, elderly population is more prone to POAF after OPCABG.^18^-^20^ Diabetes is also associated with related systemic diseases, including electrolyte disorder and metabolic syndrome, which lead to a low-grade inflammatory reaction.^21^ Stress response caused by invasive OPCABG in diabetic patients may lead to the massive release of inflammatory factors, causing metabolic syndrome and increasing the risk of POAF.^8^,^9^,^22^,^23^ Surer et al.^22^ confirmed that the incidence of POAF in OPCABG patients with high levels of glycated hemoglobin was as high as 57.1%, and logistic analysis confirmed a close relationship between hemoglobin levels and POAF in OPCABG patients. Borodashkina et al.^23^ also reported that patients with cardiovascular disease and diabetes are more prone to multivessel disease, and hyperglycemia can cause metabolic stress and coronary microvascular disease, which can also affect atrial anatomy and increase the risk of poor prognosis. These results are consistent with our observations.

In addition, our study found that a history of PCI surgery is an important risk factor for POAF after OPCABG. It is plausible that this group of patients may have a history of cardiac treatment and poor basic functional status. However, prolonged myocardial ischemia can lead to an overloaded working state of the heart, damage to the sarcoplasmic reticulum of myocardial cells, abnormal accumulation of glycogen, and excessive aggregation of cardiac collagen fibers. This, in turn, can cause atrial enlargement and degenerative fibrosis, affecting the coordination of atrial muscle activity, causing atrial dysfunction, and increasing the risk of POAF.^24^,^25^ However, Kremneva et al.^24^ found that a history of PCI treatment did not have a significant impact on the incidence of POAF and other prognostic outcomes after OPCABG. These discrepancies in the results may be related to the variability in patients’ physical condition and the quality of postoperative rehabilitation after PCI.

The current study also found that the duration of operation and the length of hospital stay are independent influencing factors for postoperative POAF in OPCABG, which is consistent with the results by Arslan et al.^26^ We may speculate that the extraction of the internal mammary artery during the surgery leads to a decrease in lung function, an increase in postoperative drainage volume, and an increase in the duration of surgery. The extraction of the internal mammary artery can also affect pleural integrity, while the placement of a thoracic drainage tube can exacerbate postoperative pain stimulation, leading to an increase in heart rate and ultimately causing POAF.^26^,^27^

In addition, Kuo et al.^28^ confirmed a close correlation between left heart function status and POAF. Güzel et al.^29^ showed that the risk of POAF is closely related to heart function status, with an incidence rate of about 10% to 20% in mild heart failure and up to about 50% in severe heart failure patients. Their study demonstrated that LVEF less than 40% was associated with the incidence of POAF as high as 29.1. On the other hand, LVEF above 50% correlated with the incidence of POAF of 23.4%, which is consistent with the results of our study.

A risk prediction model that constructed based on the identified risk factors showed good predictive value. The AUC of the current model is higher than that of the previous studies.^11^,^12^ ROC curve analysis demonstrated a relatively high diagnostic efficiency which indicates that the combined diagnosis of different types of indicators can demonstrate the predictive value of POAF after OPCABG from multiple dimensions. These findings of this current study provide evidence for a nomogram with higher predictive performance. Therefore, the constructed nomogram model in this study may allow medical staff to predict the incidence of POAF in OPCABG patients based on their scores in various items, identify high-risk OPCABG patients as early as possible, and implement certain intervention measures in a timely manner for controllable risk factors to minimize the possibility of POAF after OPCABG. Additionally, this study conducted multiple verifications on the constructed nomogram model to avoid overfitting and ensure its accuracy, and showed that the model has good predictive power for the risk of POAF after OPCABG. Therefore, it can help doctors promote more accurate management of POAF patients.

### Limitations:

Firstly, the sample size of the study is small, and all samples were taken from the same research center. Secondly, although many risk factors were included in this study, some other factors such as history of COPD and left ventricular end diastolic diameter were not included. More risk factors could be included in future studies. Thirdly, samples from other research centers were not included for external model validation. The repeatability and reliability of the nomogram model need to be validated in prospective multicenter studies with larger datasets.

## CONCLUSION

Age, diabetes, history of PCI, duration of operation, length of hospital stays, and LVEF are independent risk factors for POAF after OPCABG. The constructed nomogram model based on the identified independent risk factors has a good predictive performance. Therefore, it may be used to implement corresponding interventions in clinical practice in order to minimize the incidence of POAF and to improve the prognosis of the disease.

### Authors’ contributions:

**LQ:** Concept, study design and manuscript writing.

**CH, MF** and **ZJ:** Were involved in data collection, data analysis, interpretation and critical analysis

**LQ:** Revised the manuscript and did validation.

All authors have read, approved the final manuscript and are responsible for the integrity of the study.
